# Large Deflection of Foam-Filled Triangular Tubes under Transverse Loading

**DOI:** 10.3390/ma16093491

**Published:** 2023-05-01

**Authors:** Jianxun Zhang, Huaiyu Dong, Hao Sun, Henghui Liu, Hao Su

**Affiliations:** 1 State Key Laboratory for Strength and Vibration of Mechanical Structures, School of Aerospace Engineering, Xi’an Jiaotong University, Xi’an 710049, China; 3122106108_dhy@stu.xjtu.edu.cn (H.D.); sunhao98@stu.xjtu.edu.cn (H.S.);; 2 State Key Laboratory of Structural Analysis for Industrial Equipment, Dalian University of Technology, Dalian 116024, China; 3 Guangxi Key Laboratory of Automobile Components and Vehicle Technology, Guangxi University of Science and Technology, Liuzhou 545006, China; 4 State Key Laboratory of New Ceramic and Fine Processing, Tsinghua University, Beijing 100084, China; 5 China Nuclear Power Engineering Co., Ltd., Beijing 100840, China; suhao@cnpe.cc

**Keywords:** triangle tube, metal foam core, yield criterion, large deflection

## Abstract

In this paper, the large deflection of the foam-filled triangular tube (FFTT) is studied analytically and numerically under transverse loading. Considering the strengths of the foam and the tube, the yield criterion of FFTT is established. Based on the yield criterion, a theoretical model for the large deflection of the clamped triangular tube filled with foam under transverse loading is developed. The numerical simulations are carried out using ABAQUS/Standard software, and the analytical results are compared with the numerical ones. The effects of foam strength, thickness of the tube, and the width of the punch on the load-bearing capacity and energy absorption of the clamped FFTT loaded transversally are discussed in detail. It is demonstrated that the load-bearing ability and the energy absorption increase with increasing foam strength, tube thickness, and punch width. The closer the loading position is to the clamped end, the greater the increases in the capacity of load bearing and the energy absorption of the triangular tube filled with foam. The theoretical model can be used to foresee the large deflection of metal FFTT under transverse loading.

## 1. Introduction

The tube is a traditional energy absorbing structure, and metal foam is a lightweight material with advantages that include energy absorption, high specific stiffness, and high specific strength. Moreover, thin-walled foam-filled tube structures are widely adopted as energy-absorbing devices, owing to their high energy absorption and stable deformation. Recently, much research has focused on the energy absorption of thin-walled foam-filled tube structures with different cross-sectional shapes, including square shapes [1,2,3,4], circular shapes [5,6,7,8], octagonal shapes [9], triangular shapes [10], and other irregular shapes [11,12]. Compared with investigations of the load-bearing ability of circular and square foam-filled tubes, investigations on the load-bearing ability of triangular tubes filled with foam were few. Triangular tubes filled with foam can be used in the wings, fuselage, girders, and other structures of an aircraft, which can improve the aircraft’s impact resistance, lightness of weight, and energy absorption. Thus, it is essential to research the load-bearing ability of triangular tubes filled with foam loaded transversally.

During the past ten years, demonstrations were devoted to the investigation of the bending behavior of metal/composite tubes. For example, Huang and Zhang [13] analytically and numerically studied the impact behavior of thin-walled rectangular tubes with indentation mode under three-point bending and found that the span, wall thickness, and punch diameter have vital influences on the dynamic response of the tubes. Zhang et al. [14] experimentally and numerically researched the quasi-static bending behavior of composite multi-cell tubes and concluded that the bending resistance of an embedded multi-cell tube is around 95–130% higher than that of constituent tubes. Gupta et al. [15] conducted experimental and computational studies of the bending behavior of rectangular and square tubes made of aluminum and mild steel under quasi-static transverse loading and concluded that a square tube acquires more energy than the rectangular one. Duan et al. [16] analytically studied the bending collapse of top-hat thin-walled structures and found that they have a larger energy absorption than high-strength steel. Zheng et al. [17] researched the energy absorption of curved tubes that were subjected to lateral bending and found that the curvature has a limited effect on the energy absorption of the tubes. Wang et al. [18] investigated the bending behavior of circular–triangular nested tubes under a lateral quasi-static loading to reveal the force–deflection curves and the energy absorption performances. Sun et al. [19] analytically and numerically studied the impact behavior of tubular structures composed of different materials that were subjected to lateral loading and found that with increases in the loading angle (0°, 10°, 20°, 30°, and 45°), the impact force of aluminum tubes decreases. Nia et al. [20] numerically and analytically studied the deformation and energy absorption of thin-walled tubes with various section shapes (circular, square, rectangular, hexagonal, triangular, pyramidal, and conical) and concluded that the circular tube has the largest energy absorption capacity and the largest average force. Jia et al. [21] numerically studied the quasi-static bending behavior of composite thin-walled lenticular tubes and predicted the collapse peak moment and critical bending angle. Eyvazian et al. [22] studied the energy absorption of tubes with corrugations in various geometries that were subjected to lateral loading and found that they have a higher average impact force that is proportional to the number of corrugations and their amplitude. Meng et al. [23] researched the dynamic response of aluminum alloy tubes that were subjected to lateral impact and found that both the local indentation and the global deformation increased under higher impact energy.

In addition, investigations on the bending of tubes filled with foam have been carried out. An et al. [24] researched the crashworthiness design of a foam-filled thin-walled tube with transverse functional gradient thickness that was subjected to lateral loading and found that the tube with functionally lateral graded thickness has obvious advantages compared with tubes with traditional structures with the same weight and equal thickness. Zhu et al. [25] researched the energy absorption and deflection of thin-walled tubes with aluminum foam and carbon-fiber-reinforced plastic (CFRP) skeletons that were subjected to quasi-static lateral loading and concluded that energy absorption declines with increasing aluminum tube wall thickness and CFRP layers. Guo et al. [26] researched the large deflection of slender rectangular metal sandwich tubes filled with foam that were subjected to transverse loading, using analytical and numerical methods. Zhang et al. [27] analytically and numerically studied the large deflection of a slender circular metal tube filled with foam that was subjected to lateral loading and found that the load and the absorbed energy increased with the width of the punch, the metal foam strength, and the ratio of metal foam thickness to wall thickness. Yin et al. [28] analytically and numerically investigated the bending behavior of multi-cell thin-walled structures filled with foam that were subjected to lateral loading and determined the optimal design of thin-walled structures. Mantena et al. [29] studied the flexural strength of composite square steel tubes filled with polyurethane foam and found that the load-bearing ability of composite square steel tubes filled with polyurethane foam improved more than tubes made of empty steel. Zhang et al. [30] investigated the bending performance of improved Al matrix syntactic foam-filled circular tube and found that the bending resistance and energy absorption performance of foam-filled tubes are mainly controlled by wall thickness and foam. Duarte et al. [31] evaluated the potential use of integral-skin foams as stiffening elements for aluminum alloy thin-walled tubes under quasi-static and dynamic bending and found that the filling foam led to an upgraded bending response of the foam-filled tubes that was greater than the sum of the bending responses for the independent components. Fang et al. [32] demonstrated the bending behavior of functionally foam-filled tubes and concluded that functionally graded foam-filled tubes can produce better Pareto solutions than their common uniform-foam counterpart.

To the authors’ knowledge, investigations on the plastic behavior of triangular foam-filled tubes under transverse loading have been few. The main aim of this paper is to research the large deflection of triangular tubes filled with foam under lateral loading, analytically and numerically. This paper is arranged as follows. In Section 2, the statement of the problem is presented. In Section 3, the plastic yield criterion of triangular tubes filled with foam is established. In Section 4, the analytical solution for the plastic behavior of triangular tubes filled with foam under lateral loading is derived. In Section 5, the finite element analysis is conducted. In Section 6, the analytical results are compared with the numerical ones and the effects of the thickness of the tube, the strength of the foam, and the width of the punch on the plastic behavior of triangular tubes filled with foam are discussed in detail. Concluding remarks are presented in Section 7.

## 2. Problem Formulation

**Figure 1:**
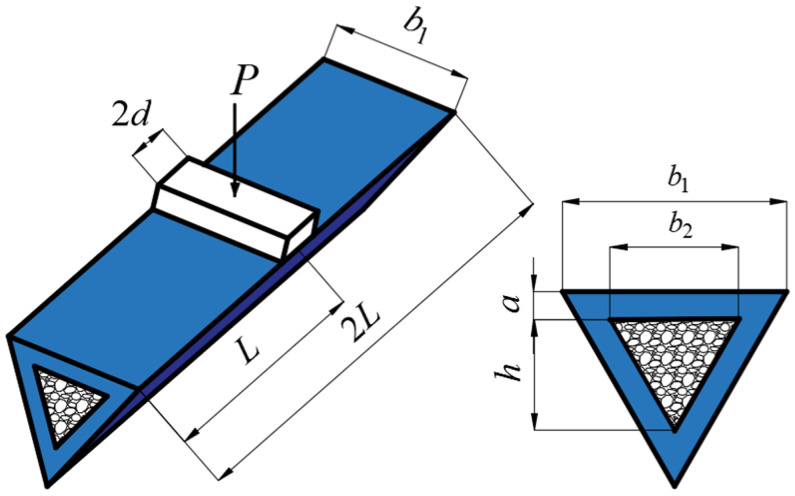
The schematic diagram of a clamped triangular tube filled with foam, subjected to transverse loading.

Consider a metal slender equilateral triangular tube filled with foam that is subjected to lateral loading *P* by a flat punch at midspan, in which the length of the triangular tube is 2*L*, the width of the foam is *b*_2_, the height of the foam is *h*, the thickness of the tube is *a*, the height of the tube is 3*a* + *h*, and the width of the punch is 2*d*, as depicted in Figure 1. It is supposed that the metal tube obeys rigid plastic material with yield strength *σ_f_*, and the metal foam obeys rigid-perfect-plastic-locking material with yield strength $\sigma_{c}$ and densification strain $\varepsilon_{D}$.

## 3. Yield Criterion of a Foam-Filled Triangular Tube

It is well known that for a symmetric structure, the plastic neutral surface and the geometric neutral surface coincide. Conversely, the plastic neutral surface and the geometric neutral surface are not coincident in an asymmetric structure. Here, the physical neutral surface is defined as $z_{p}$. It can be calculated from the following equation:

(1) $$\int_{-\frac{2}{3}h-a_{2}}^{z_{p}}\sigma (z)dz=\int_{z_{p}}^{\frac{1}{3}h+a_{1}}\sigma (z)dz,$$

where σ(z) indicates the yield stress of the metal foam-filled triangular tube section.

Then,

(2) $$z_{p}=\frac{-4h\sigma_{c}-12a\sigma_{f}+3\sqrt{2}\sqrt{h^{2}{\sigma_{c}}^{2}+a^{2}\sigma_{c}\sigma_{f}+6ah\sigma_{c}\sigma_{f}+8a^{2}{\sigma_{f}}^{2}}}{6\sigma_{c}},$$

Here, there are two different situations for $z_{p}$, i.e., in the lower part of the tube and in the foam. Then, $\bar{z}$ can be defined as

(3) $$\bar{z}=\frac{-4\bar{\sigma }-12\bar{a}+3\sqrt{2}\sqrt{{\bar{\sigma }}^{2}+{\bar{a}}^{2}\bar{\sigma }+6\bar{a}\bar{\sigma }+8{\bar{a}}^{2}}}{2\bar{\sigma }},$$

where $\bar{\sigma }=\frac{\sigma_{c}}{\sigma_{f}},$ $\bar{a}=\frac{a}{h}$.

**Figure 2:**
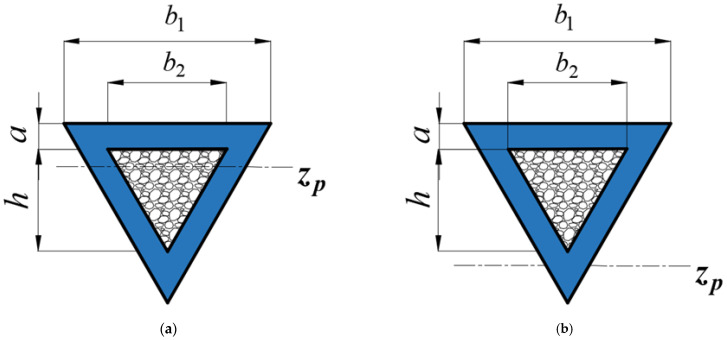
The location of the plastic neutral surface of a triangular tube filled with foam. (**a**) $\bar{z}\in [0,1]$, and (**b**) $\bar{z}\in [1,+\infty ]$.

When $\bar{z}\in [0,1]$, the plastic neutral surface locates in the metal foam, as shown in Figure 2a. When $\bar{z}\in [0,+\infty ]$, the plastic neutral surface locates in the lower part of the metal tube, as shown in Figure 2b. Here, $\bar{z}\in [0,1]$ is considered.

**Figure 3:**
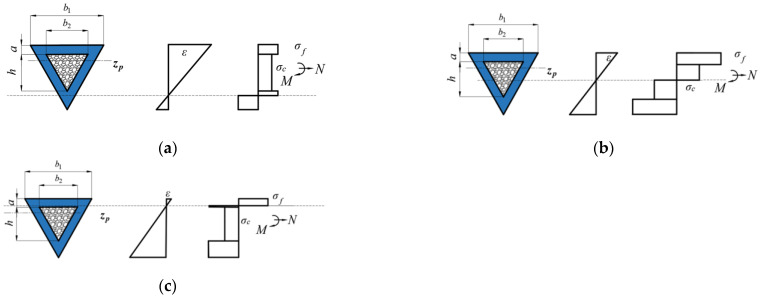
Strain and stress distributions of a triangular foam-filled tube section. (**a**) $0<x<\frac{2a}{3a+h}$ (**b**) $\frac{2a}{3a+h}<x<\frac{2a+h}{3a+h}$ (**c**) $\frac{2a+h}{3a+h}<x<1$.

The distance from the plastic neutral surface to the bottom surface of the foam-filled triangular tube is defined as *H*, which can be expressed as $H=x(a_{1}+a_{2}+h)$. Figure 3 displays the strain and stress distributions of the triangular foam-filled tube section.

Thus, the axial force and the bending moment of the triangular tube filled with foam are described as

(4) $$N=\left\{ \begin{array}{l} \frac{1}{2}[hb_{2}\sigma_{f}(\frac{\sigma_{c}}{\sigma_{f}}-1)-(-1+2x^{2})Db_{1}\sigma_{f}],\;\;\;\;\;\;\;\;\;\;0<x<\frac{2a}{3a+h}\\ \frac{1}{2}\{h-2[x(1+3\frac{a}{h})-2\frac{a}{h}]2\}b_{2}\sigma_{f}(\frac{\sigma_{c}}{\sigma_{f}}-1)+\frac{1}{2}D(1-2x^{2})b_{1}\sigma_{f},\frac{2a}{3a+h}<x<\frac{2a+h}{3a+h}\\ \frac{1}{2}[-hb_{2}\sigma_{f}(\frac{\sigma_{c}}{\sigma_{f}}-1)-(-1+2x^{2})Db_{1}\sigma_{f}],\;\;\;\;\;\;\;\;\;\frac{2a+h}{3a+h}<x<1 \end{array}\right.$$

(5) $$M=\left\{ \begin{array}{l} \frac{b_{2}}{h}\sigma_{f}D^{2}x^{2}[z_{p}-\frac{2}{3}D(x-1)]-\frac{1}{2}\frac{b_{2}}{h}z_{p}\sigma_{f}E,\;\;\;\;\;\;0<x<\frac{2a}{3a+h}\\ {}[-\frac{2}{3}(Dx-H)+z_{p}](Dx-2a)2\sigma_{c}\frac{b_{2}}{h}-\frac{1}{2}\sigma_{c}\frac{b_{2}}{h}z_{p}h^{2}\\ -\frac{b_{2}}{h}\frac{8}{3}a^{2}\sigma_{f}H+\frac{b_{2}}{h}a\sigma_{f}Dx(\frac{8}{3}D+4z_{p})-\frac{1}{2}\frac{b_{2}}{h}z_{p}a\sigma_{f}G-2a\frac{b_{2}}{h}\sigma_{f}D^{2}x^{2},\\ \frac{2a}{3a+h}<x<\frac{2a+h}{3a+h}\\ \frac{b_{2}}{h}\sigma_{f}D^{2}x^{2}[z_{p}-\frac{2}{3}D(x-1)]-\frac{1}{2}\frac{b_{2}}{h}z_{p}\sigma_{f}F-\frac{b_{2}}{h}z_{p}\sigma_{f}h^{2},\\ \frac{2a+h}{3a+h}<x<1 \end{array}\right.$$

where D=3a+h, $E=3a(3a+2h)+h^{2}\frac{\sigma_{c}}{\sigma_{f}},$ $F=-3a(3a+2h)+h^{2}\frac{\sigma_{c}}{\sigma_{f}},$ G=17a+6h, H=2a+h.

When *x* = 0, *x* =$\frac{z_{p}+a_{2}+\frac{2}{3}h}{h+a_{1}+a_{2}}$, the maximum axial force *N_A_* and the bending moment *M_A_* of the triangular tube filled with foam cross-section are

(6) $$N_{A}=\frac{1}{2}[hb_{2}(\sigma_{c}-\sigma_{f})+(3a+h)b_{1}\sigma_{f}],$$

and

(7) $$\begin{array}{l} M_{A}=\frac{8}{81}h^{2}b_{2}\sigma_{c}+\frac{1}{18}z_{p}\frac{b_{2}}{h}\sigma_{c}[-h^{2}+6z_{p}(2h+z_{p})]\\ \;\;\;+\frac{8}{9}a\frac{b_{2}}{h}\sigma_{f}(3a^{2}+3ah+h^{2})+\frac{1}{6}a\frac{b_{2}}{h}\sigma_{f}z_{p}(-3a-2h+12z_{p}), \end{array}$$

Then, Equations (6) and (7) can be expressed as

(8) $$\frac{N_{A}h}{M_{A}}=\frac{b_{2}(\bar{\sigma }-1)+(3\bar{a}+1)b_{1}}{\frac{1}{81}(I_{1}+9I_{2})},$$

where $\bar{b_{1}}=\frac{b_{1}}{h},\;$$\bar{b_{2}}=\frac{b_{2}}{h},\;$$\bar{z_{p}}=\frac{z_{p}}{h},\;$$A=-1+6\bar{z_{p}}(2+\bar{z_{p}}),\;$$B=1+3{\bar{a}}^{2}+3\bar{a},\;$$C=-2+12\bar{z_{p}}-3\bar{a},$ $I_{1}=\bar{\sigma }(16+9A\bar{z_{p}}),$ $I_{2}=\bar{a}(16B+3\bar{z_{p}}C)$.

Then, from Equations (4)–(8), the expression of the yield criterion of triangular tubes filled with foam can be described as

(9) $$n=\left\{ \begin{array}{l} \frac{\bar{b_{2}}(\bar{\sigma }-1)-(2x^{2}-1)(3\bar{a}+1)\bar{b_{1}}}{\bar{b_{2}}(\bar{\sigma }-1)+(3\bar{a}+1)\bar{b_{1}}},\;\;\;\;\;\;\;\;\;0<x<\frac{2\bar{a}}{3\bar{a}+1}\\ \frac{\{1-2{\left[x(3\bar{a}+1)-2\bar{a}\right]}^{2}\}\bar{b_{2}}(\bar{\sigma }-1)+(3\bar{a}+1)(-2x^{2}+1)\bar{b_{1}}}{\bar{b_{2}}(\bar{\sigma }-1)+(3\bar{a}+1)\bar{b_{1}}},\;\frac{2\bar{a}}{3\bar{a}+1}<x<\frac{2\bar{a}+1}{3\bar{a}+1}\\ \frac{\bar{b_{2}}(-\bar{\sigma }+1)-(2x^{2}-1)(3\bar{a}+1)\bar{b_{1}}}{\bar{b_{2}}(\bar{\sigma }-1)+(3\bar{a}+1)\bar{b_{1}}},\;\;\;\;\;\;\;\;\frac{2\bar{a}+1}{3\bar{a}+1}<x<1 \end{array}\right.$$

(10) $$m=\left\{ \begin{array}{l} \frac{27\{2{\bar{D}}^{2}x^{2}[3\bar{z_{p}}-2\bar{D}(x-1)]-3\bar{z_{p}}\bar{E}\}}{I_{1}+9I_{2}},\;\;\;\;\;\;\;\;\;0<x<\frac{2\bar{a}}{3\bar{a}+1}\\ \frac{-27\bar{\sigma }\{4[\bar{D}x-\bar{H}-6\bar{z_{p}}]{\left[\bar{D}x-2\bar{a}\right]}^{2}+3\bar{z_{p}}\}}{I_{1}+9I_{2}}\\ -\frac{27\bar{a}\{4[4\bar{a}\bar{H}-2\bar{D}(2\bar{D}+3\bar{z_{p}})x+3{\bar{D}}^{2}x^{2}]+3\bar{z_{p}}\bar{G}\}}{I_{1}+9I_{2}},\;\;\;\frac{2\bar{a}}{3\bar{a}+1}<x<\frac{2\bar{a}+1}{3\bar{a}+1}\\ \frac{27\{2{\bar{D}}^{2}x^{2}[3\bar{z_{p}}-2\bar{D}(x-1)]-3\bar{z_{p}}(\bar{F}-2)\}}{I_{1}+9I_{2}},\;\;\;\;\;\;\;\frac{2\bar{a}+1}{3\bar{a}+1}<x<1 \end{array}\right.$$

where $\bar{D}=3\bar{a}+1,$ $\bar{E}=\bar{\sigma }+3\bar{a}(3\bar{a}+2),$ $\bar{F}=\bar{\sigma }-3\bar{a}(3\bar{a}+2),$ $\bar{G}=17\bar{a}+6,$ $\bar{H}=2\bar{a}+1$.

**Figure 4:**
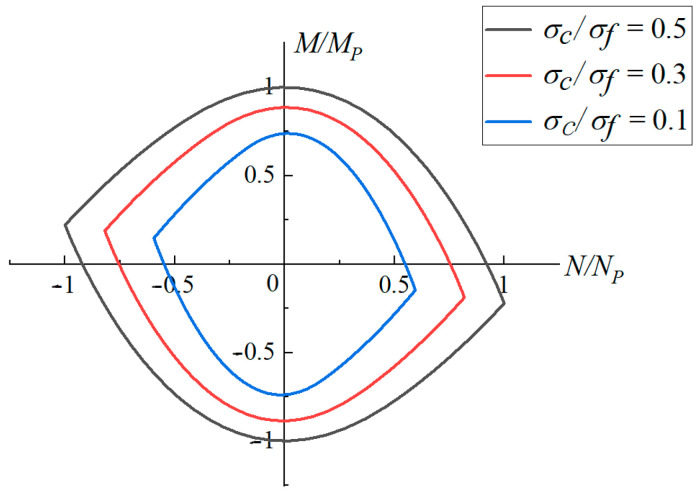
Yield loci for the triangular foam-filled tube section with various foam strengths ($\bar{a}=\frac{1}{3}$, $\bar{b_{2}}=\frac{\sqrt{3}}{2}$, $\bar{b_{1}}=\frac{\sqrt{3}}{4}$).

The yield surfaces of metal triangular tubes filled with various foam strengths are shown in Figure 4, in which $\bar{a}=\frac{1}{3}$, $\bar{b_{2}}=\frac{\sqrt{3}}{2}$, $\bar{b_{1}}=\frac{\sqrt{3}}{4}$. It can be seen that when the yield strength of the metal foam is lower, the yield surface is closer to the origin. 

## 4. Analytical Solution

**Figure 5:**
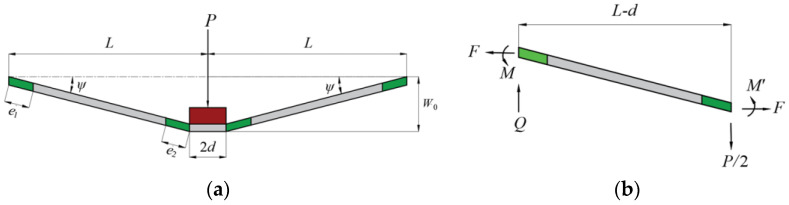
Global deformation of plastic neutral surface of the triangular tube filled foam under lateral loading. (**a**) Deflection, (**b**) force and bending moment.

Consider a clamped slender triangular tube filled with foam that is subjected to transverse loading by a flat punch with a width of 2*d* at midspan, in which the length of the tube is 2*L*, as depicted in Figure 1. It is assumed that overall bending occurs in triangular tubes filled with foam, and local denting below the punch can be neglected. The maximum deflection below the punch is *W*_0_ and the angle at the two ends of the triangular tube filled with foam is *ψ*, as shown in Figure 5.

The total elongation of the left part is *e* and the elongations at the end and loading position are *e*_1_ and *e*_2_; then,

(11) $$e=e_{1}+e_{2}$$

The total angular rotation can be expressed as

(12) $$\psi \approx \frac{W_{0}}{L-d}$$

The total elongation can be expressed as

(13) $$e\approx \frac{W_{0}{}^{2}}{2(L-d)}$$

According to the associated flow rule of the plastic yield criterion, the end and loading position on the left part of the metal foam-filled triangular tube can be described as

(14) $$\frac{N_{A}}{M_{A}}\frac{{\dot{e}}_{1}}{\dot{\psi }}=\frac{N_{A}}{M_{A}}\frac{{\dot{e}}_{2}}{\dot{\psi }}=-\frac{\frac{d\phi }{d(\frac{N}{N_{A}})}}{\frac{d\phi }{d(\frac{N}{N_{A}})}}=-\frac{dM}{dN}\frac{N_{A}}{M_{A}}$$

From Equations (11) to (14), the bending deflection *W*_0_ of the metal triangular tube filled with foam can be described as

(15) $$W_{0}=-2\frac{dM}{dN}$$

Then,

(16) $$W_{0}{}^{*}=\frac{W_{0}}{3a+h}=\left\{ \begin{array}{l} -\frac{2\bar{b_{2}}[(3x-2)\bar{D}-3\bar{z_{p}}]}{3\bar{b_{1}}},\;\;\;\;\;\;\;\;\;\;\;\;0<x<\frac{2\bar{a}}{3\bar{a}+1}\\ \frac{2\bar{b_{2}}\{(2\bar{a}-x\bar{D})[-3x\bar{D}+2(\bar{a}+\bar{H}+3\bar{z_{p}})]\bar{\sigma }+2\bar{a}[(3x-2)\bar{D}-3\bar{z_{p}}]\}}{[3\bar{b_{2}}(2\bar{a}-x\bar{D})(\bar{\sigma }-1)-3x\bar{b_{1}}]\bar{D}},\\ \frac{2\bar{a}}{3\bar{a}+1}<x<\frac{2\bar{a}+1}{3\bar{a}+1}\\ -\frac{2\bar{b_{2}}[(-3x+2)\bar{D}+3\bar{z_{p}}]}{3\bar{b_{1}}},\;\;\;\;\;\;\;\;\;\;\;\;\frac{2\bar{a}+1}{3\bar{a}+1}<x<1 \end{array}\right.$$

According to the equilibrium equation,

(17) $$M+M^{\prime }+FW_{0}-\frac{P}{2}(L-d)=0$$

where F≈N. The global deformation of the metal triangular tube filled with foam occurs when N=0, $M=M_{A}$, $W_{0}=0$. A limit load *P*_0_ can be determined by

(18) $$P_{0}=\frac{4M_{A}}{L-d}$$

From Equations (8) to (10) and (19), the relationship between the normalized load P^*^ and the normalized deflection $W_{0}{}^{*}$ can be expressed as

(19) $$P^{*}=\frac{P}{P_{c}}=\frac{m+\frac{1}{2}nW_{0}{}^{*}\frac{\bar{b_{2}}(\bar{\sigma }-1)+(3\bar{a}+1)\bar{b_{1}}}{\frac{1}{81}\bar{b_{2}}(I_{1}+9I_{2})}\bar{D}}{1-\bar{d}}$$

where $P_{c}=\frac{4M_{p}}{L}$, $\bar{d}=\frac{d}{L}$.

During the whole deformation process, the work is equal to the plastic energy of the triangular tube filled with foam. Then,

(20) $$U=\int_{0}^{W_{0}}P(W_{0})dW_{0}$$

and

(21) $$U^{*}=\frac{U}{P_{c}(3a+h)}$$

## 5. Finite Element Analysis

Using ABAQUS/Implicit software, the plastic behavior of clamped triangular tubes filled with foam that are subjected to lateral loading is numerically studied in this section. The metal tubes and foam were modeled by three-dimensional eight-node linear brick elements (Type C3D8R) with reduced integration. The punch was modeled as rigid, and deformation was applied to the punch. The metal foam was filled in the metal tube. The interaction between tube and foam was defined as “Tie”. The symmetric boundary condition was imposed on the section of the tube at its midspan. All degrees of freedom at both ends of the tube were set to zero. There was no frictionless contact set between the rigid punch and the triangular tubes filled with foam. The surface between the bottom of the punch and the top of the tube was established in order to set up the contact. The historical variables were set to the displacement at the midpoint of the tube, the interaction force between the tube and the punch, and the energy generated by the tube under lateral loading, as were the field variables.

The thickness of the metal tube was *a* = $\frac{10\sqrt{3}}{3}$ mm, the width of which was *b*_1_ = 40 mm, the foam width was *b*_2_ = 20 mm, the foam height was *h* = $10\sqrt{3}$ mm, and the punch width was 2*d* = 30 mm. Two cases of the length of the tube were considered. i.e., 2*L* = 600 mm (Case 1) and 1200 mm (Case 2). The metal tubes obeyed the *J*_2_ plastic flow theory, which is based on an aluminum alloy with density *ρ_f_* = 2700 kg/m^3^, Young’s modulus *E_f_* = 69 GPa, elastic Poisson’s ratio 0.3, yield stress $\sigma_{f}$ = 400 MPa, and the strain hardening modulus *E_t_* = $4\times 10^{-4}E_{f}$. A Deshpande–Fleck constitutive model [33] was used to calculate the plastic behavior of the metal foam. The metal foam was made of aluminum with Young’s modulus *E_c_* =1 GPa, yield strength *σ_c_* = 9.8 MPa, elastic Poisson’s ratio *ν_ce_* = 0.3, density ρ = 270 kg/m^3^, densification strain *ε_D_* = 0.5, and linear strengthening modulus *E_tc_* = 12*E_c_* beyond the densification. It was assumed that the metal tubes and the foam had sufficient strength and toughness, without fracture. The mesh sensitivity check of the numerical results showed that the numerical results with the additional mesh refinement were not significantly different from the present numerical results.

## 6. Conclusions and Discussion

**Figure 6:**
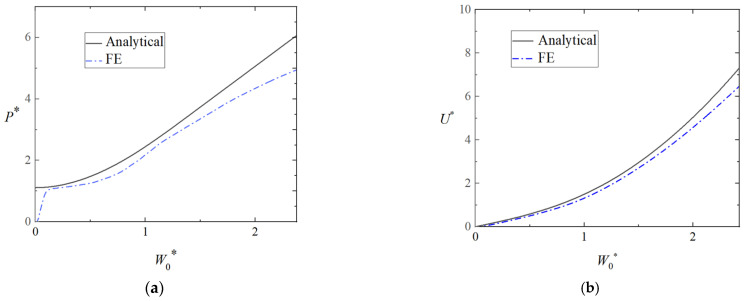
Comparison of theoretical and numerical results for the plastic behavior of the clamped triangular tube filled with foam (Case 1). (**a**) Load-deflection curves and (**b**) energy-deflection curves.

Figure 6a,b provides a comparisons of theoretical and numerical load-deflection and energy-deflection curves of triangular tubes filled with foam (Case 1) loading at midspan, in which $\frac{a}{h}=\frac{1}{3}$, $\frac{b_{2}}{h}=\frac{2}{\sqrt{3}}$, and $\frac{b_{1}}{h}=\frac{4}{\sqrt{3}}$. It can obviously be noticed that the analytical results are in good agreement with the numerical ones. 

**Figure 7:**
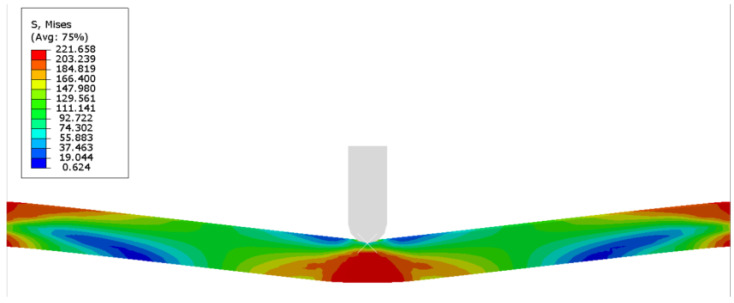
Von Mises stress distribution of the triangular tube filled with foam under transverse loading at midspan.

There were differences between the analytical results and the numerical results. The curve of the analytical results was higher than that of the numerical ones in small deflections, as the elastic property was neglected in the analytical model. In addition, the shear force was not considered in the analytical model. The analytical energy-deflection curves coincided well with the numerical ones, which were a little higher than the numerical ones. It can be obviously noticed, as shown in Figure 7, that the local denting below the punch was not evident (Case 1).

**Figure 8:**
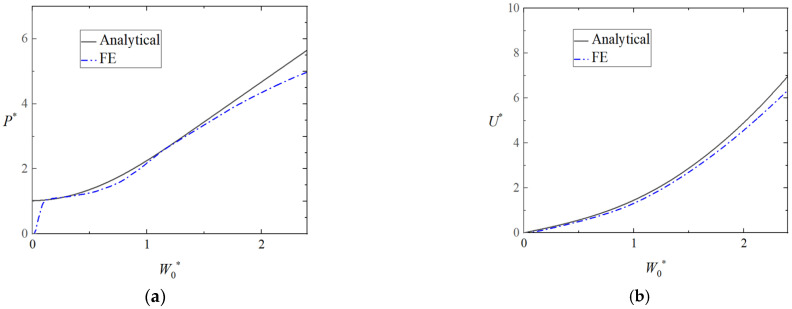
Comparison of theoretical and numerical results for the plastic behavior of the clamped triangular tube filled with foam (Case 2). (**a**) Load-deflection curves and (**b**) energy-deflection curves.

In addition, Figure 8a,b provides a comparison of the theoretical and numerical load-deflection and energy-deflection curves of triangular tubes filled with foam (Case 2) loading at midspan. It can be seen that the analytical results agree well with the numerical ones. 

**Figure 9:**
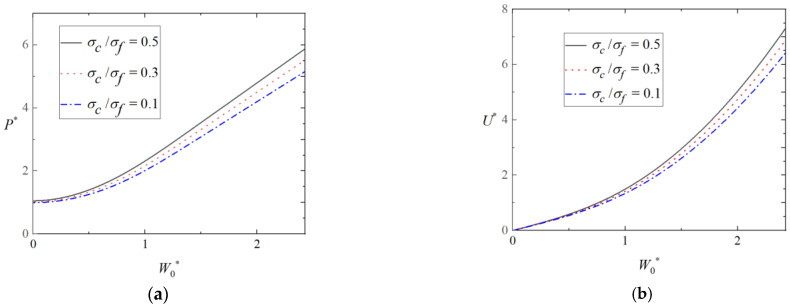
Effect of foam strength on plastic behavior of triangular tubes filled with foam. (**a**) Load-deflection curves and (**b**) energy-deflection curves.

Figure 9a,b shows the effect of foam strength on the load-deflection curves and energy-deflection curves of the triangular tubes, in which $\frac{a}{h}=\frac{1}{3}$, $\frac{b_{2}}{h}=\frac{2}{\sqrt{3}}$, $\frac{b_{1}}{h}=\frac{4}{\sqrt{3}}$, and $\bar{d}$ = 0.05. As can be seen from Figure 9a, for the given deflection, the load-bearing ability and energy absorption of the triangular tube significantly improves with increases in the foam strength.

**Figure 10:**
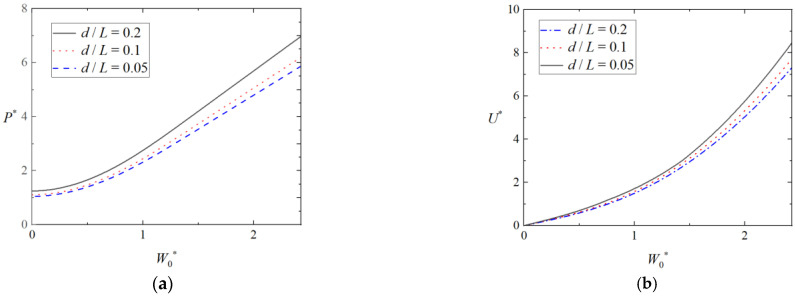
Effect of punch width on plastic behavior of triangular tubes filled with foam. (**a**) Load-deflection curves and (**b**) energy-deflection curves.

Figure 10a,b shows the effect of punch width on the load-deflection curves and energy-deflection curves of triangular tubes filled with foam, in which $\frac{a}{h}=\frac{1}{3}$, $\frac{b_{2}}{h}=\frac{2}{\sqrt{3}}$, $\frac{b_{1}}{h}=\frac{4}{\sqrt{3}}$, and $\bar{\sigma }=0.5$. The load and energy increase with increasing punch width. According to Equation (19), the wider the flat punch becomes, the larger the lateral load. 

**Figure 11:**
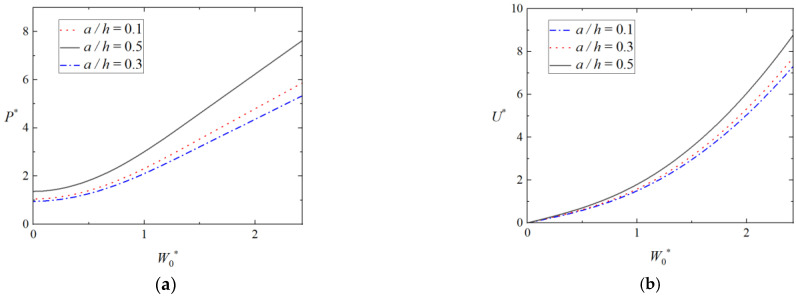
Effect of tube thickness on plastic behavior of triangular tubes filled with foam. (**a**) Load-deflection curves and (**b**) energy-deflection curves.

Figure 11a,b shows the effect of the tube thickness on load-deflection curves and energy-deflection curves of the foam-filled tubes, in which $\frac{b_{2}}{h}=\frac{2}{\sqrt{3}}$, $\frac{b_{1}}{h}=\frac{4}{\sqrt{3}}$,$\bar{\sigma }=0.5$, $\bar{d}$ = 0.05. For the given deflection, the energy absorption and load of triangular tubes filled with foam increases when the thickness of tube increases. It is demonstrated that the tube thickness makes a significant affection on the plastic behavior of the triangular foam-filled tube.

## 7. Conclusions

The large deflection of clamped metal triangular tubes filled with foam that were subjected to lateral loading was studied analytically and numerically. Considering the strength of the tubes and the foam, the yield criterion of a metal triangular tube filled with foam was established. On basis of the yield criterion, a theoretical model to analyze the plastic behavior of the clamped triangular tube filled with foam was established, in which the relation between stretching and the bending moment was considered. The theoretical results agreed well with the numerical ones. It was demonstrated that the load-bearing ability and energy absorption increased with increasing foam strength, tube thickness, and punch width. The closer the loading position is to the clamped end, the greater the increases in the ability of load bearing and the energy absorption of the triangular tube filled with foam. The present theoretical model can be used to foresee the large deflection of metal triangle tubes filled with foam under lateral loading.

## Data Availability

The authors confirm that all data for this study are included in the paper.
